# Thulium Laser Resection of Bladder Tumors vs. Conventional Transurethral Resection of Bladder Tumors for Intermediate and High Risk Non-Muscle-Invasive Bladder Cancer Followed by Intravesical BCG Immunotherapy

**DOI:** 10.3389/fsurg.2021.759487

**Published:** 2021-11-08

**Authors:** Zheng Liu, Gongwei Long, Yucong Zhang, Guoliang Sun, Wei Ouyang, Shen Wang, Hao Xu, Zhihua Wang, Wei Guan, Xiao Yu, Zhiquan Hu, Zhong Chen, Shaogang Wang, Heng Li

**Affiliations:** ^1^Department of Urology, Tongji Medical College, Tongji Hospital, Huazhong University of Science and Technology, Wuhan, China; ^2^Hubei Institute of Urology, Tongji Medical College, Tongji Hospital, Huazhong University of Science and Technology, Wuhan, China; ^3^Department of Geriatrics, Tongji Medical College, Tongji Hospital, Huazhong University of Science and Technology, Wuhan, China

**Keywords:** thulium laser, en bloc resection of bladder tumor, non-muscle-invasive bladder cancer, bacillus Calmette-Guérin vaccine, transurethral resection of bladder tumors

## Abstract

**Background:** Thulium laser resection of bladder tumors (TmLRBT) is recently considered as a common treatment option for non-muscle-invasive bladder cancers (NMIBC), but whether it is superior to Transurethral resection of bladder tumors (TURBT) are still undetermined.

**Materials and Methods:** We retrospectively screened our institution database to identify patients who were treated by conventional TURBT or TmLRBT for NMIBC and followed by intravesical bacillus Calmette-Guérin (BCG) immunotherapy. The preoperative characteristics, perioperative outcomes, and recurrence-free survival were compared to assess the safety and efficacy of the two procedures.

**Results:** Eventually, 90 patients who underwent TmLRBT (*n* = 37) or TURBT (*n* = 53) followed by intravesical BCG immunotherapy were included. Two groups were similar in baseline characteristics except for the smaller tumor size of the TmLRBT group(1.7 cm vs. 2.2 cm; *P* = 0.036). Obturator nerve reflex occurred in eight patients in the TURBT group and 3 of them suffered from bladder perforation while none happened in the TmLRBT group. The TmLRBT also had a shorter irrigation duration. In the multivariate Cox regression, the TmLRBT was related to less recurrence risk (HR: 0.268; 95% CI, 0.095–0.759; *P* = 0.013).

**Conclusion:** Our results suggested that TmLRBT is safer than conventional TURBT with fewer perioperative complications, and it offers better cancer control, therefore might be a superior option for NMIBC patients with intermediate and high recurrence risk.

## Introduction

Bladder cancer ranks second common urological malignancy worldwide (1). It represents a spectrum of diseases, from non-muscle-invasive bladder cancer (NMIBC), which is defined as the tumor confined to the bladder mucosa or submucosa, to invasiveand advanced diseases that demand aggressive treatment. Approximately 75% of newly diagnosed bladder cancer is NMIBC (2).

The conventional transurethral resection of bladder tumor (TURBT) is the most common strategy for NMIBC and it is recommended by the guidelines (3, 4). However, the TURBT has a complication rate of ~4–6%, of which urinary tract infections and significant haematuria are most common (5). In some cases, major complications including obturator nerve reflex (ONR) and bladder perforation could occur. To overcome these drawbacks, lasers including holmium YAG and thulium YAG were introduced. Several studies have suggested the superior safety of Thulium laser resection of bladder tumors (TmLRBT) compared with conventional TURBT (6, 7).

The TmLRBT is increasingly used in the treatment of NMIBC recently, but whether it can provide better cancer control than TURBT is still unclear. Previous studies have compared the recurrence rates of these two therapies but no significant difference was detected (7). However, in all these studies, intravesical therapies were conducted using epirubicin or mitomycin C, instead of bacillus Calmette-Guérin (BCG), which is superior for preventing the recurrence of NMIBC (8–10). Here we retrospectively collected the data of patients who underwent TmLRBT or TURBT followed by BCG therapy to assess the safety and efficacy of these two therapies.

## Materials and Methods

The study and all its protocols were approved by the institutional review board of the Tongji Hospital, Tongji Medical College, Huazhong University of Science and Technology (Grant number: TJ-IRB20210106). The informed consent was exempted for this retrospective and observational study. All data has been de-identified.

We accessed our institutional database to retrospectively screened all the patients who were treated by conventional TURBT or TmLRBT from August 2018 to December 2019. The inclusion criteria were as follows. (1) Pathologically confirmed as NMIBC. (2) Underwent conventional TURBT or TmLRBT. (3) Intermediate or high risk according to EAU risk stratification (11). (4) Received standard BCG intravesical therapy. (5) With complete clinical and follow-up data. The exclusion criteria were as follows. (1) Locally advanced (T2 or higher), or metastatic bladder cancers. (2) Loss of contact or inadequate clinical information for further analysis. (3) Unable to finish BCG intravesical therapy due to intolerance or other reasons. (4) Comorbidity of other neoplastic diseases.

The medical records of all patients were retrieved and the baseline characteristics were collected. Ultrasonography, intravenous urography, computerized tomography of urinary system (CTU), and cystoscopy were routinely performed before resection to assess the clinical characteristics of the tumors. All the patients chose TmLRBT or TURBT after being informed of the advantages and drawbacks of the two surgical procedures and signed the informed consent. All the surgeries were performed according to standard protocols which have been described in our previous study (12).

If postoperative gross hematuria occurred after surgery, continuous bladder irrigation would be maintained until no sign of postoperative bleeding for 4 h. 30 mg gemcitabine was used for intravesical instillation therapy within 24 h after surgery for the first time.

For patients with intermediate or high risk (11), intravesical BCG therapy would be recommended. Two weeks after that, according to the drug instructions, 2 g BCG in 50 ml of saline was given weekly for 6 weeks, then biweekly for 6 weeks, and then once a month for 10 months. For high-risk patients, monthly intravesical instillations were added for 1–2 years. The ultrasonography and cystoscopy were performed every 3 months for the first 2 years after surgery for recurrence surveillance. An additional telephone follow-up was conducted for patients who performed examinations in local medical institutions.

The statistical analysis was conducted using the SPSS 20.0 software. Continuous data were presented as mean ± standard deviation and compared using Student's *t*-test. Categorical data were presented as number (percentage) and compared with the Chi-square or Fisher exact test. Univariate Cox regressions were used to evaluate the predictive role of covariates, including surgery type, age, gender, previous bladder tumor, second resection, tumor number, tumor size, tumor location, pathologic stage, and pathologic grade for recurrence-free survival (RFS). Variables with a *P*-value <0.1 were furtherly included in multivariate Cox regression. The Kaplan-Meier(K-M) curve of RFS was plotted and the log-rank test was conducted with the Graphpad Prism 8.0.1 software.

## Results

Eventually, 37 patients who underwent TmLRBT and 57 patients who conducted TURBT group were enrolled in the analysis. As listed in Table 1, the baseline characteristics such as age, gender, tumor number, tumor stage, and pathological grade were similar between two groups. The tumor size of the TmLRBT is smaller than that of the TURBT group (1.7 cm vs. 2.2 cm; *P* = 0.036). Nine patients in the TmLRBT group and 6 patients in the TURBT group have a history of bladder tumor and the proportion was comparable in these two groups.

**Table 1:** Characteristics of included patients and tumors.

**Variable**	**TmLRBT (*n* = 37)**	**TURBT (*n* = 53)**	***P*-value**
Age, year	60.6 ± 9.2	61.2 ± 11.6	0.780
Gender			0.830
Male	30 (81.1%)	42 (79.2%)	
Female	7 (18.9%)	11 (20.8%)	
Previous bladder tumor	9 (24.3%)	6 (11.3%)	0.103
Tumor number	2.9 ± 2.9	2.3 ± 4.0	0.406
Tumor multiplicity			0.520
Single	17 (45.9%)	28 (52.8%)	
Multiple	20 (54.1%)	25 (47.2%)	
Tumor size, cm	1.7 ± 0.8	2.2 ± 1.1	**0.036**
Tumor Location			0.607
Lateral	27 (73.0%)	36 (67.9%)	
Other	10 (27.0%)	17 (32.1%)	
T stage			0.525
Ta	16 (43.2%)	20 (37.7%)	
Tis	3 (8.1%)	2 (3.8%)	
T1	18 (48.6%)	31 (58.5%)	
Tumor Grade (WHO2004)			0.341
PUNLMP	0	2 (3.8%)	
Low Grade	8 (21.6%)	11 (20.8%)	
High Grade	29 (78.4%)	40 (75.5%)	
Risk			
Intermediate	6 (16.2%)	12 (22.6%)	0.453
High	31 (83.8%)	41 (77.4%)	

*Data presented as n (%) or mean ± SD*.

*P-value < 0.05 was considered statistically significant and highlighted in bold*.

*TmLRBT, Thulium laser resection of bladder tumors; TURBT, Transurethral resection of bladder tumors; TIS, tumor in situ; PUNLMP, papillary urothelial neoplasms of low malignant potential*.

The perioperative results of the two groups are illustrated in Table 2. The operation duration of the two groups was similar. During the TURBT, 8 (15.1%) patients encountered ONR, and 3 (5.7%) patients had bladder perforation. Meanwhile, no ONR or bladder perforation occurred during the TmLRBT surgery. After surgery, only one patient in the TURBT group experienced TUR syndrome, and no second surgery for hemostasis was conducted. Postoperative gross hematuria happened in 51 (96.2%) patients in the TURBT group and 16 (43.2%) patients in the TmLRBT group. 50 patients in the TURBT group and 12 patients in the TmLRBT group received postoperative irrigation. Among these patients who underwent postoperative irrigation, the TmLRBT group also has a shorter irrigation duration (6.5 h vs. 24.3 h; *P* < 0.001) than conventional TURBT. Besides, the TmLRBT could shorten the catheterization time (2.2 d vs. 3.1 d; *P* = 0.002).

**Table 2:** Intraoperative, postoperative and oncological outcomes.

**Variable**	**TmLRBT (*n* = 37)**	**TURBT (*n* = 53)**	***P*-value**
Operative time, min	31.4 ± 17.2	39.4 ± 23.2	0.075
Obturator nerve reflex	0	8 (15.1%)	**0.015**
Bladder perforation	0	3 (5.7%)	0.381
TUR syndrome	0	1 (1.9%)	1.000
Post-operative gross hematuria	16 (43.2%)	51 (96.2%)	** <0.001**
Post-operative irrigation	12 (32.4%%)	50 (94.3%)	** <0.001**
Duration of irrigation^*^, h	6.5 ± 4.9	24.3 ± 13.7	**<0.001**
Second surgery for hemostasis	0	0	1.000
Post-operative catheterization, d	2.2 ± 0.7	3.1 ± 1.7	**0.002**
Second resection	7 (18.9%)	4 (7.5%)	0.189
Recurrence within 3 months	0	3 (5.7%)	0.381
Recurrence within 1 year	3 (8.1%)	15 (28.3%)	**0.018**

*Data presented as n (%) or mean ± SD*.

*P-value < 0.05 was considered statistically significant and highlighted in bold*.

*TmLRBT, Thulium laser resection of bladder tumors; TURBT, Transurethral resection of bladder tumors*.

* *Only the data of patients underwent irrigation was analyzed*.

**Figure 1:**
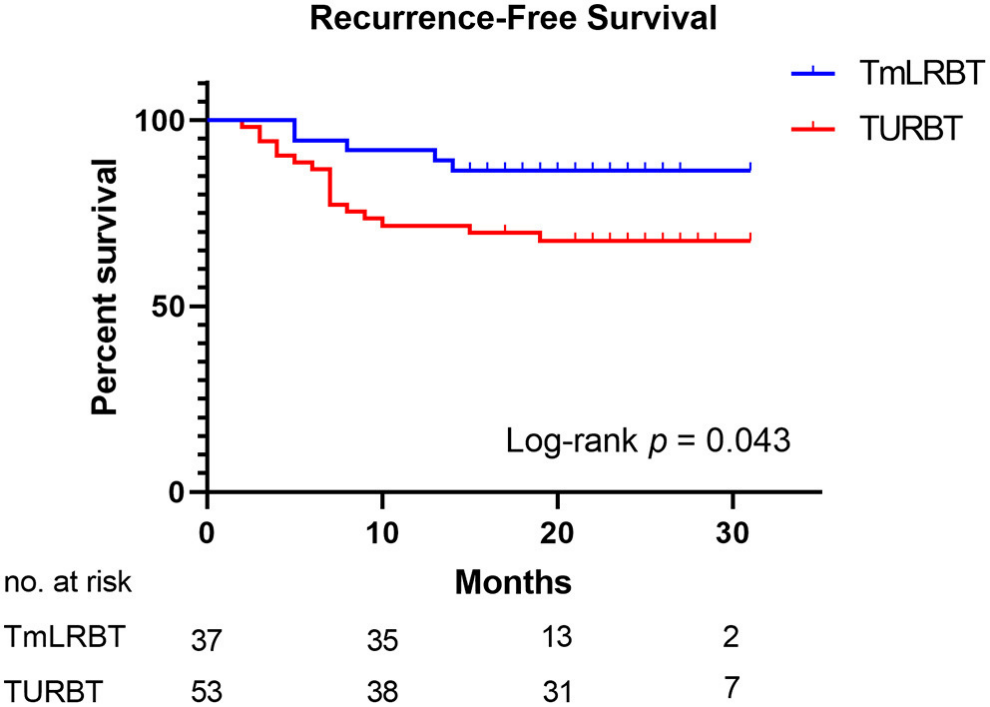
The K-M curve of recurrence-free survival of the patients who underwent TmLRBT or TURBT.

The RFS graph was illustrated in Figure 1 and the K-M curve of RFS showed that the TmLRBT group has a longer RFS than the TURBT group (HR: 0.376; 95% CI, 0.162–0.873; Log-rank *P* = 0.043). Univariate and multivariate Cox regressions were conducted to evaluate the predictive value of variates and the results were shown in Table 3. As the multivariate Cox analysis suggested, the surgery type (HR: 0.268; 95% CI, 0.095–0.759; *P* = 0.013), history of bladder tumor (HR: 4.319; 95% CI, 1.733–10.769; *P* = 0.002), and pathologic stage (HR: 3.033; 95% CI, 1.023–8.997; *P* = 0.045) are independent predictive factors of RFS. Notably, 3 patients who underwent TURBT experienced recurrence within 3 months after the surgery.

**Table 3:** Univariate and multivariate Cox analyses of recurrence-free survival.

**Variable**	**Univariate analysis**		**Multivariate analysis**	
	**HR (95% CI)**	***P-*value**	**HR (95% CI)**	***P*-value**
Surgery (Ref: TURBT)	0.376 (0.138–1.019)	0.054	0.268 (0.095–0.759)	**0.013**
Second resection (Ref: No)	0.040 (0.001–7.682)	0.229		
Gender (Ref: Female)	0.829 (0.306–2.247)	0.712		
Age (Ref: <70 year)	2.190 (0.807–5.942)	0.124		
Previous bladder tumor (Ref: No)	3.372 (1.407–8.079)	**0.006**	4.319 (1.733–10.769)	**0.002**
Tumor number (Ref: single tumor)	1.517 (0.648–3.551)	0.337		
Tumor size (Ref: <3cm)	0.540 (0.160–1.826)	0.540		
Tumor location (Ref: other)	1.127 (0.441–2.882)	0.803		
Pathologic stage (Ref: Ta)	3.482 (1.177–10.303)	**0.024**	3.033 (1.023–8.997)	**0.045**
Pathologic grade (Ref: PUNLMP and low grade)	2.029 (0.600–6.862)	0.255		
Risk (Ref: Intermediate risk)	2.698 (0.630–11.550)	0.181		

*P-value < 0.05 was considered statistically significant and highlighted in bold*.

*Variables with a p-value < 0.1 in the univariate analysis were included in the multivariate analysis*.

*TURBT, Transurethral resection of bladder tumors; PUNLMP, papillary urothelial neoplasms of low malignant potential*.

## Discussion

Currently, TURBT combined with intravesical therapy is still the “gold standard” for intermediate and high risk NMIBC, and BCG immunotherapy is the recommended adjuvant therapy by guidelines (3, 4). Compared with intravesical chemotherapy including epirubicin and mitomycin C, BCG immunotherapy showed better efficacy in recurrence prevention (9, 10). However, in the previous studies comparing the prognosis of TmLRBT and TURBT, BCG immunotherapy was not applied to these patients (13–18). To the best of our knowledge, the present study is the first research to compare the efficacy of TmLRBT and TURBT under BCG intravesical therapy. In this study, over 90% (89 of 90) patients completed intravesical BCG therapy for 1 year. The completion of each groups was summarized in Table 4.

**Table 4:** Summary of the BCG therapy completion.

	**TmLRBT**	**TURBT**
Abortion	4 (10.8%)	4 (7.5%)
Completion of 1 year BCG therapy	29 (78.4%)	41 (77.4%)
Completion of 2 year BCG therapy	3 (8.1%)	5 (9.4%)

*TmLRBT, Thulium laser resection of bladder tumors; TURBT, Transurethral resection of bladder tumors*.

Cancer control is the most critical purpose in the treatment of malignancies. The spreading of tumor cells caused by TURBT could be a potential reason for recurrence and progression (19–22). During the TmLRBT procedures, the en bloc technique allows a complete enucleation of lesions and avoid tumor fragmentation. Therefore it can potentially minimize the amount of floating tumor cells and diminish the risk of dissemination (23). However, the previous studies did not suggest a significant advantage of TmLRBT in cancer control. In our analysis, after a standard BCG immunotherapy, the TmLRBT showed superior efficacy in the prevention of recurrence than TURBT, which is quite different from the results of most previous studies, in which postoperative intravesical chemotherapy was applied. On the one hand, compared with previous researches, our research included more patients with pathologic high grades (76.7% vs. 10.0% to 30.0%), T1 or Tis stages(60.0% vs. 26.4% to 48.6%), and most patients were high risk (80%) (14–16, 18). The benefit of TmLRBT might be more significant in these tumors with higher risk. On the other hand, the use of BCG immunotherapy might reinforce cancer control of TmLRBT and both treatments synergically suppressed the recurrence. For these tumors with signs of aggressive properties in the preoperative assessments, TmLRBT might be a preferred option.

The complications of TURBT are an essential concern in the treatment of NMIBC. During the TURBT procedure, especially for tumors locating at the lateral bladder wall, the current flow may stimulate the obturator nerve and lead to muscle contraction. In some cases, it could even bring bladder perforation. Several techniques were developed to prevent ONR during the TURBT, including the use of bipolar electrodes and the obturator nerve block. However, the efficacy of bipolar electrodes in the prevention of ONR is still controversial (24–26). As for the obturator nerve block, to achieve a higher success rate, the assistance of ultrasound or nerve stimulator might be needed and the procedure could be time-consuming and complicated. While in the TmLRBT, no current flow was produced and the ONR and bladder perforation could be perfectly avoided.

Another advantage of TmLRBT is the excellent performance in hemostasis. Under the thulium laser, the exposed tissue could be vaporized after being heated to a temperature of 90–100°C. As for the tissue adjacent to the vaporized part, it could be coagulated under 60–80°C (27). The instantly coagulated tissue layer made the hemostasis more efficient. Several studies suggested that TmLRBT was related to a lower postoperative irrigation rate (14) and a shorter irrigation length (15, 16). In our study, fewer postoperative gross hematuria and a lower postoperative irrigation rate in the TmLRBT group were also observed. Even for these patients who need irrigation, the TmLRBT had a shorter irrigation time. Compared with conventional TURBT, the TmLRBT is more feasible for NMIBC.

Notably, thulium laser is not the only laser used in the resection of bladder cancers. Studies had suggested the safety and efficacy of different lasers to treat NMIBC including holmium laser (6), green-light lithium triborate laser (28), and potassium-titanyl-phosphate laser (29). Thulum laser has a wavelength of 2 um, which is nearing the absorption peak of water, therefore it has the most efficient vaporization (30). Another advantage of thulium laser is its shallowest penetration depth compared to holmium and green-light laser (31), which allows more accurate resection and might reduce the risk of bladder perforation.

This present study has several limitations. The limited sample size is the main drawback of this study. As BCG immunotherapy was not widely used in China until recent years, most patients received maintain chemotherapy such as gemcitabine and epirubicin instead of BCG therapy in the past. Also, the retrospective nature of this study might bring potential bias. For example, in our study, we included these patients who received BCG therapy after surgery, which resulted in a significantly higher proportion of patients with pathologic high grades. The significant oncological outcomes alert us to conduct subgroup analysis in future prospective research to assess the efficacy of TmLRBT. The selection bias during the therapy determination should also be noted. The tumor characteristics could affect the therapy choosing. For example, the TURBT could be often used when handling large size tumors, which could be reflected in the imbalanced tumor size in Table 1. Also, the surgeon's preference could also affect the final resection strategy. Besides, as a retrospective study, all cases were collected from the clinical practice. The clinicians managing post-operative care were aware of the surgical methods of each patient. The postoperative parameters such as catheterization and irrigation duration could be biased.

## Conclusion

In summary, our results suggested that TmLRBT is safer than conventional TURBT with fewer perioperative complications. Besides, TmLRBT could offer better cancer control, therefore might be a superior option for NMIBC patients with intermediate and high recurrence risk. The findings of our study should be ascertained in a further prospective study with larger sample size and longer follow-up.

## Data Availability Statement

The original contributions presented in the study are included in the article/Supplementary Material, further inquiries can be directed to the corresponding authors.

## Ethics Statement

The studies involving human participants were reviewed and approved by Institutional Review Board of the Tongji Hospital, Tongji Medical College, Huazhong University of Science and Technology (Grant Number: TJ-IRB20210106). Written informed consent for participation was not required for this study in accordance with the national legislation and the institutional requirements.

## Author Contributions

ZL and GL wrote this manuscript. YZ, GS, WO, SheW, and HX collected the data. GL analyzed the data. ZW, WG, XY, ZH, and ZC read and edited the manuscript. ShaW and HL designed the study. All authors approved the submitted version.

## Funding

This work is supported by the Natural Science Fund of Hubei Province (Grant Number: 2018CFB459, Recipient: HL). The funding sources had no role in the design and conduct of the study, collection, management, analysis, and interpretation of the data, and preparation, review, or approval of the manuscript.

## Conflict of Interest

The authors declare that the research was conducted in the absence of any commercial or financial relationships that could be construed as a potential conflict of interest.

## Publisher's Note

All claims expressed in this article are solely those of the authors and do not necessarily represent those of their affiliated organizations, or those of the publisher, the editors and the reviewers. Any product that may be evaluated in this article, or claim that may be made by its manufacturer, is not guaranteed or endorsed by the publisher.

## Supplementary Material

Figure S1 Diagram for the BCG intravesical therapy schedule.
