# Supplementary figures and images for: Thulium Laser Resection of Bladder Tumors vs. Conventional Transurethral Resection of Bladder Tumors for Intermediate and High Risk Non-Muscle-Invasive Bladder Cancer Followed by Intravesical BCG Immunotherapy
Source: Front Surg. 2021 Nov 8;8:759487. doi: 10.3389/fsurg.2021.759487 (PMC8606824; doi:10.3389/fsurg.2021.759487)

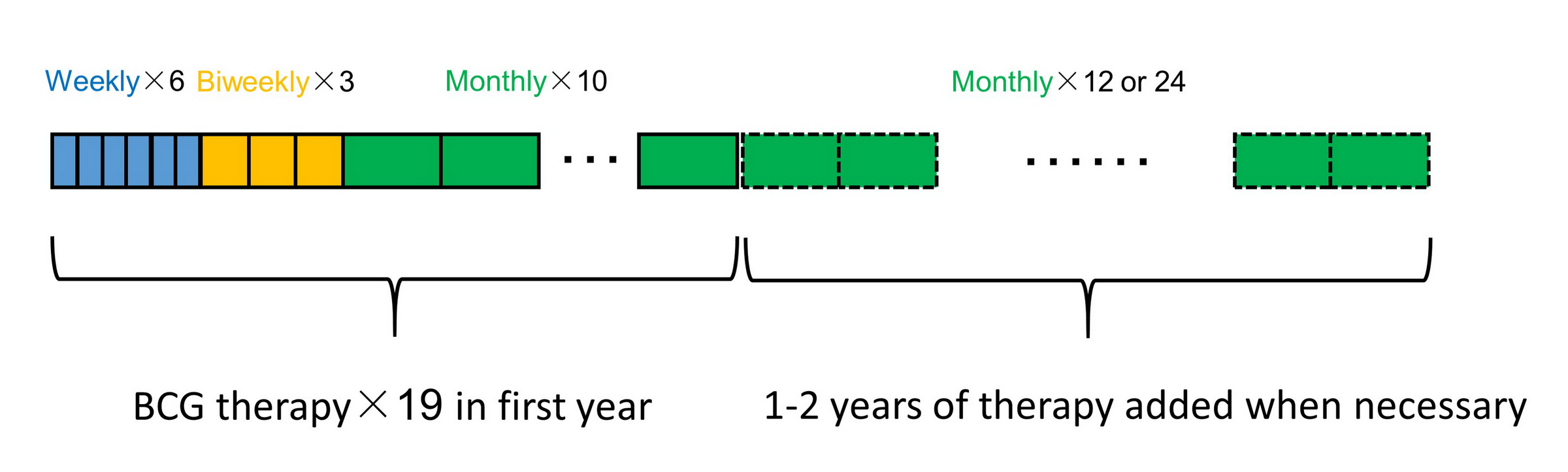

Supplement: Figure S1 — Diagram for the BCG intravesical therapy schedule. [file Image_1.TIF]
